# Detecting sarcasm in multi-domain datasets using convolutional neural networks and long short term memory network model

**DOI:** 10.7717/peerj-cs.645

**Published:** 2021-08-25

**Authors:** Ramish Jamil, Imran Ashraf, Furqan Rustam, Eysha Saad, Arif Mehmood, Gyu Sang Choi

**Affiliations:** 1 Khwaja Fareed University of Engineering and Information Technology, Rahim Yar Khan, Pakistan; 2 Information and Communication Engineering, Yeungnam University, Gyeongsan si, Daegu, South Korea; 3 The Islamia University of Bahawalpur, Bahawalpur, Pakistan

**Keywords:** Sarcasm detection, Multi-domain sarcastic comments, Convolutional neural networks, Social media, Long short term memory network

## Abstract

Sarcasm emerges as a common phenomenon across social networking sites because people express their negative thoughts, hatred and opinions using positive vocabulary which makes it a challenging task to detect sarcasm. Although various studies have investigated the sarcasm detection on baseline datasets, this work is the first to detect sarcasm from a multi-domain dataset that is constructed by combining Twitter and News Headlines datasets. This study proposes a hybrid approach where the convolutional neural networks (CNN) are used for feature extraction while the long short-term memory (LSTM) is trained and tested on those features. For performance analysis, several machine learning algorithms such as random forest, support vector classifier, extra tree classifier and decision tree are used. The performance of both the proposed model and machine learning algorithms is analyzed using the term frequency-inverse document frequency, bag of words approach, and global vectors for word representations. Experimental results indicate that the proposed model surpasses the performance of the traditional machine learning algorithms with an accuracy of 91.60%. Several state-of-the-art approaches for sarcasm detection are compared with the proposed model and results suggest that the proposed model outperforms these approaches concerning the precision, recall and F1 scores. The proposed model is accurate, robust, and performs sarcasm detection on a multi-domain dataset.

## Introduction

Social media has emerged as one of the most influential platforms to express opinions, views, emotions, and information. Consequently, a huge amount of data is generated each day from social platforms like Twitter, Facebook and Instagram, etc. A large number of corporate organizations and governments utilize this data to perceive and analyze general population sentiments about a specific person, idea, product, or entity. So, the sentiment analysis on the social media data has gained a large interest recently. Sentiment analysis focuses on the identification of the polarity using the sentiments or emotions from the data into various classes such as positive, negative, and neutral. Sentiment polarity determines users’ response towards a product which helps the entrepreneurs to take preventive and corrective measures to meet their standards and demands. Similarly, criticism of public service helps the governments to analyze public needs and make important decisions (Gautam & Yadav, 2014).

On account of its importance, a large body of works on sentiment analysis has been presented. However several challenges require further investigation, one of which is sarcasm detection. It is important to recognize literal, as well as, figurative meanings in the opinions posted on social platforms. Sarcasm implies that various positive words and emotions are posted in tweets that represent negative, slang, or undesirable characteristics (Sarsam et al., 2020). Sarcasm is a means to convey negative emotions or opinions using positive or aggravating words (Kumar et al., 2020). For instance, ‘I love working here for nothing’ is a sarcastic sentence where ‘love’ is used as irony or mockery to criticize the working environment. Unlike humans who can understand its meaning easily, the use of positive words makes it very challenging for machine learning approaches to understand the figurative nature of the sarcasm in text. As a result, sarcasm can switch the polarity of a tweet from negative to positive if not dealt with properly (Liebrecht, Kunneman & Van Den Bosch, 2013). Sarcasm detection is considered one of the most challenging tasks in sentiment analysis since it is difficult to determine the intensity, sharpness, and pitch of voice in textual data, which often helps to understand sarcasm. The significance of sarcasm detection for sentiment analysis and its challenges makes it one of the emerging research problems (Joshi, Bhattacharyya & Carman, 2017).

Several models for sarcasm detection have been presented that incorporate statistical, machine learning, and rule-based approaches but predominantly they utilize simple datasets (Mandal & Mahto, 2019; Pozzi et al., 2016). However, such approaches are not capable of perceiving the figurative meaning of words (Joshi, Bhattacharyya & Carman, 2017). Furthermore, these approaches require handcrafted features and are unable to understand the patterns in passive voice sentences (Bajwa & Choudhary, 2006). However, instead of utilizing handcrafted features, approaches incorporating deep neural networks (DNNs) learn the imperative features automatically. These networks show results similar to human experts due to the logical structure which allows them to analyze the data recurrently (LeCun, Bengio & Hinton, 2015). Deep learning approaches tend to show superior performance than traditional machine learning approaches for sarcasm detection (Abdi et al., 2019).

Sarcasm has become a norm in verbal and non-verbal communications and has been studied on a wide scale by linguistics, behavioral scientists, and psychologists. A wide range of theories describing the process of sarcasm has been explored (Rajadesingan, Zafarani & Liu, 2015). A rich variety of automated sarcasm detection approaches have been presented utilizing various datasets including long texts, short texts, dialogues, and transcripts (Joshi, Bhattacharyya & Carman, 2017). However, when it comes to sarcasm detection in multi-domain data, it remains an under-researched area and a challenging task (Pang & Lee, 2008). This study proposes a distinctive deep learning-based detection of sarcasm using multi-domain data and utilizes a hybrid approach of convolution neural network (CNN) and long short-term memory layer (LSTM). The main contributions of the current study can be summarized as

- A novel approach is presented for sarcasm detection using the multi-domain data. The proposed approach uses CNN for feature extraction while the LSTM is used for training and testing.
- Three features are investigated for their suitability and efficacy including term frequency-inverse document frequency (TF-IDF), a bag of words (BoW), and global vectors (GloVe) for word representations.
- Several traditional machine learning algorithms are included in the study to evaluate the performance of the proposed approach. Machine learning algorithms include decision tree (DT), random forest (RF), extra tree classifier (ETC), and support vector machine (SVM) and these are tested with both the TF-IDF and BoW separately.
- Performance of the proposed approach is compared with several state-of-the-art approaches on sarcasm detection for evaluating its efficacy. Results demonstrate the superior performance of the proposed approach.

The rest of the paper is organized in the following fashion. The Related Work section discusses the research works which are closely related to the current study. Material and Methods contain the description of the proposed approach and its working methodology. The dataset used for experiment setup, performance metrics, and results is given in the Experiment and Results section. In the end, the conclusion and future works are given.

## Related work

Sarcasm detection has been a mainstream research area over the last few years due to the popularity of social media platforms and microblogging websites. Consequently, a wide range of research works can be found in the literature that focuses on sarcasm detection from text data.

For the most part, sarcasm detection has been performed on the textual data by exploring the pragmatic and lexical features. For example, the authors incorporate the chi-square test to shortlist the extracted features which helps enhance the performance of a voting classifier for sarcasm detection in Gupta et al. (2020). Similarly, Kumar & Garg (2019) perform sarcasm detection in typo-graphic memes of Instagram posts using the lexical-based supervised techniques integrated with pragmatic and semantic features. Khatri, Pranav & Anand (2020) point out that word embedding elevates the performance of a model higher than the traditional feature extraction techniques. In the light of these findings, several works utilize the pragmatic and lexical features for sarcasm detection.

An ensemble model is proposed in Lemmens et al. (2020) where long short-term memory (LSTM), CNNC-LSTM, SVM, and multilayer perceptron (MLP) are utilized together. The model incorporates GloVe features for sarcasm detection in social media reviews. The reported results are promising with higher accuracy than traditional machine learning algorithms. The performance of deep learning-based models can be enhanced using multi-head attention as reported in Kumar et al. (2020) where a BiLSTM is used to detect sarcasm. Similarly, bidirectional encoder representations transformers (BERT) can increase the sarcasm detection accuracy for Twitter and Reddit tweets. For this purpose, the correlation among the response and context dialogue sequence is extracted by combining the aspect-based sentiment with BERT. Javdan, Minaei-Bidgoli & Atai (2020) proposed a hybrid approach comprising the convolution neural network (convNet) and soft attention-based bidirectional long short-term memory (sAtt-BLSTM). Two features are merged including GloVe and punctuation-based auxiliary features for sarcasm detection in tweets.

Several other research works utilize deep learning models for sarcasm detection in reviews and tweets. For example, the authors perform sarcasm detection in Majumder et al. (2019) using GRU-based neural networks on the sentiments from the text data. The research shows that there is a strong correlation between sentiments and sarcasm. Various attributes of sarcastic expressions are captured in Ren et al. (2020) with the help of sentiment semantics for sarcasm detection on Twitter and internet argument *corpus* (IAC-V1, IAC-V2) datasets. The performance of the CNN models is enhanced using a local max-pooling layer instead of the traditional max-pooling layer. Results suggest that the contrast of sentiment, contextual information, and local information can provide higher accuracy for sarcasm detection. The accuracy of the sarcasm detection can be improved by including the last utterance in a dialogue chain as reported by Baruah et al. (2020). The study leverages the large version of the BERT classifier with 16 attention heads and sigmoid activation layers for sarcasm detection and achieves higher accuracy than traditional machine and deep learning approaches. Similarly, the authors present a hybrid approach in Hazarika et al. (2018) where content-based modeling contextual sarcasm detector (CASCADE) is used with the stylometric embedding of the users on a large scale Reddit dataset. The study suggests that user embedding integrated with discourse features plays an important role to increase the accuracy of sarcasm detection. In the same fashion, Ren, Ji & Ren (2018) reports that incorporating the features of the tweets and contextual features in word vector enhances the efficacy of sarcasm detection.

Two similar works that use deep learning models include Agrawal & An (2018) and Son et al. (2019). Agrawal & An (2018) propose effective word embedding for sarcasm (AWES) and uses it on six datasets including forum posts, reviews and tweets. The proposed model combines effective knowledge with contextual information where the BiLSTM is used to capture the contextual information and LSTM to capture effective information. The study concludes that for long texts and short texts, the emotions and effective word representations, respectively, tend to show higher accuracy. Son et al. (2019) introduce sAtt-BLSTM convNet, a deep learning model that is based on the hybrid of sAtt-BLSTM and convNet. Results indicate that incorporating GloVe for word representation and building semantic word embedding improves the accuracy of sarcasm detection on random tweets.

Besides the detection process of sarcastic words, several approaches evaluate and present features that can enhance sarcasm detection. For example, Lunando & Purwarianti (2013) utilize features of interjection functions with negativity evaluation and the lexical phenomenon of the tweets to improve sarcasm detection. The authors use behavioral traits from Twitter data for sarcasm detection in Rajadesingan, Zafarani & Liu (2015). The features derived from different forms of sarcasm such as text expression-based features, familiarity-based features, complexity-based features, and contrast-based features tend to show higher performance. The set of features is divided concerning the sarcastic behaviors such as features based on complexity, expression of emotions, contrast and familiarity. Similarly, Amir et al. (2016) report that the extensive set of features that are drafted carefully from the dataset shows robust performance. The authors report that conventional approaches can not capture the subtle form of context incongruity in sarcasm detection (Joshi et al., 2016). Sarcasm detection can be improved by semantic similarities in word embedding with unweighted similarity features and distance-weighted similarity features. The research states that the fusion of features extracted by content word and function word corresponds to better sarcasm detection (Mukherjee & Bala, 2017). Similarly, the use of context incongruity (the incompatibility of text) is reported to produce good results for sarcasm detection in Joshi, Sharma & Bhattacharyya (2015).

In addition to the already discussed research works, a detailed discussion of sarcastic tweets involving figurative language can be found in Abulaish, Kamal & Zaki (2020). The survey paper discusses various categories of figurative language involving tweets like sarcasm, irony, and satire, etc., and presents an in-depth discussion of each category. Furthermore, state-of-the-art machine and deep learning approaches are presented for the detection of tweets involving figurative language. Characterizing features of each category, open-access datasets, and computational approaches to automatically detect these tweets are discussed in detail.

From the literature discussed above, it can be concluded that most of the studies under the umbrella of sarcasm detection utilize word embedding and other feature extraction techniques on traditional datasets to achieve improved accuracy. A summary of the discussed research works is provided in Table 1. Moreover, the existing research works focus on sarcasm detection using single domain datasets where the training and testing involve domain-specific tweets or reviews. On the other hand, this study advances sarcasm detection from single domain to multi-domain dataset which is a complex task.

**Table 1:** A summary of the research works on sarcasm detection.

Reference	Dataset	Methods	Findings
(Gupta et al., 2020)	Tweets	Combined the punctuation and sentiment related features with top 200 features extracted by TF-IDF for a voting classifier	Extraction and elimination of punctuation and sarcastic features enhances the accuracy of sarcasm detection.
(Kumar & Garg, 2019)	Typo-graphic Memes	Incorporated semantic, lexical, and pragmatic features with KNN, decision tree, support vector classifier (SVC) with RBF kernel and linear kernel, random forest (RF), and multiLayer perceptron (MLP).	Hand-crafted features help to enhance the performance of the MLP with typo-graphic memes.
(Khatri, Pranav & Anand, 2020)	Tweets	GloVe and BERT embedding with logistic regression, SVM, RF, and Gaussian Naïve (GN).	Efficiency of sarcasm detection is elevated by incorporating embedding.
(Lemmens et al., 2020)	1. Tweets 2. Reddit Comments	Ensemble of adaboost classifier integrated with decision tree as base estimator, learning probabilities of sarcasm predicted by four component models including LSTM, MLP, CNN- LSTM , and SVM.	Sarcasm detection on Reddit data is intrinsically more challenging.
(Kumar et al., 2020)	Reddit Comments	Bidirectional Long Short-Term Memory integrated with multi-head attention (MHA-BiLSTM).	Incorporating multi-head attention-based system in BiLSTM improves the sarcasm detection accuracy.
(Javdan, Minaei-Bidgoli & Atai, 2020)	1. Tweets 2. Reddit Comments	Several models including NBSVM, BERT, BERT-SVM, BERT-LR, XLNET, Bi-GRU-CNN+BiLSTM-CNN, IAN, LCF-BERT, and BERT-AEN	Models pre-trained with a combination of BERT and aspect-based sentiment analysis enhances the performance of sarcsdm detection.
(Son et al., 2019)	Tweets	A hybrid of soft attention-based LSTM and CNN	Semantic word embeddings from GloVe assists helps to show robustness for sarcasm detection.
(Jena, Sinha & Agarwal, 2020)	1. Tweets 2. Reddit Comments	Contextual-Network (C-Net)	Sarcastic nature of a conversation can be efficiently captured by integrating the context.
(Majumder et al., 2019)	Text Snippets	GRU-based neural network	There is a correlation between sentiment and sarcasm of the context.

## Materials and Methods

This study proposes a framework comprising of convolutional neural network (CNN) and long short term memory (LSTM) network. The framework is based on a deep neural network that can emulate the biological neurons and perform complex computational modeling. Deep neural network (DNN) incorporates artificial neurons joined together and share their output with the conceding neurons. Comprising of input, hidden and output layers, they also include an optimization or a loss function to optimize the output (Li, Hua & Wu, 2020). The weights are improved with each repetition to improve the desired output.

### 1D convolutional neural network

**Figure 1:**
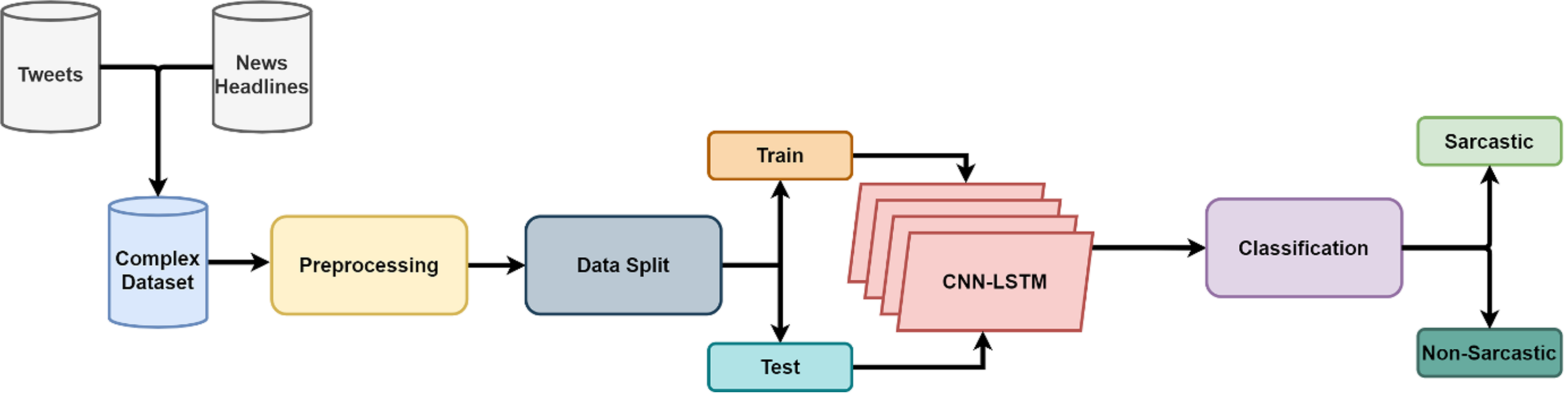
Operation of one dimensional convolutional neural network before passing features to LSTM.

One-dimensional convolution networks use weights to learn from the input (Wang, Mao & Li, 2020). The dot product is performed on the input received by each neuron connected to the network. Training of the CNN requires relatively a smaller number of weights in comparison to fully-connected structure making it easy to use (Eren, Ince & Kiranyaz, 2019). A 1D CNN is shown in Fig. 1 in which *X*_1_, *X*_2_, *X*_3_,…,*X*_*n*_ are the inputs and *F*_1_, *F*_2_, … ,*F*_*n*_ are the features that are mapped by 1D convolutional layer. Green, blue and red is the connections bearing their weight values through which the input layer is connected to the convolutional layer. Each connection of the same color has the same weight values.

### Long short term memory network

**Figure 2:**
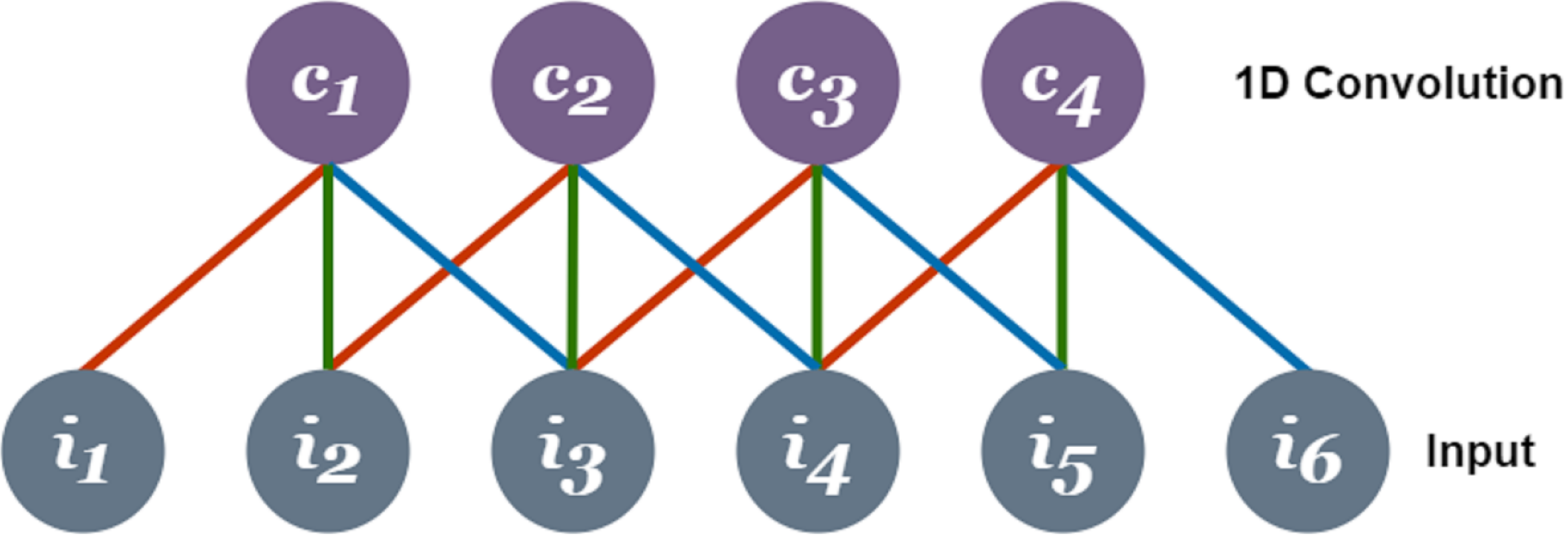
Process of LSTM where, *α* = sigmoid function, *x_t_* = input, *h_t_* −1 = previous state, *s_t_* = statevariable, *s_t_* − 1 = state variable lagging one-time stamp and *h_t_* = output.

A recurrent neural network (RNN) is a form of DNNs that keeps track of the information related to what computations have been performed so far by integrating feedback cycles (Yu et al., 2019). The RNN incorporates a temporary memory to store the context information for a short time. It considers the current input and hidden state that prevents it to map long-term dependencies if the gap between two-time steps becomes too long (Ghosh & Veale, 2016). This inability of RNNs is overcome by the introduction of LSTM where a set of input gate and output gate collectively decides the output as a function of the previous state *h*_(_*t* − 1) and input *x*_*t*_ at each time step (Zhou et al., 2015). The input gate lets the *tanh* function assign each input value weight between −1 and 1 concerning its significance and the sigmoid function (*α*) to decide which values to keep (1) or omit (0). Furthermore, the forget gate omits the information from the cell using the decision taken by the sigmoid function (*α*) in a specific timestamp using the hidden state (*h*_*t*_ − 1) and input (*x*_*t*_). Whereas, the part of the cell that makes the output is decided by the output gate (Shahid, Zameer & Muneeb, 2020). Another phenomenon that dominates LSTM over RNNs is its effective capability to learn with longer time stamps and counter gradient vanishing in the backpropagation (Si et al., 2019). Figure 2 shows the process of a typical LSTM network.

### Proposed CNN-LSTM model

**Figure 3:**
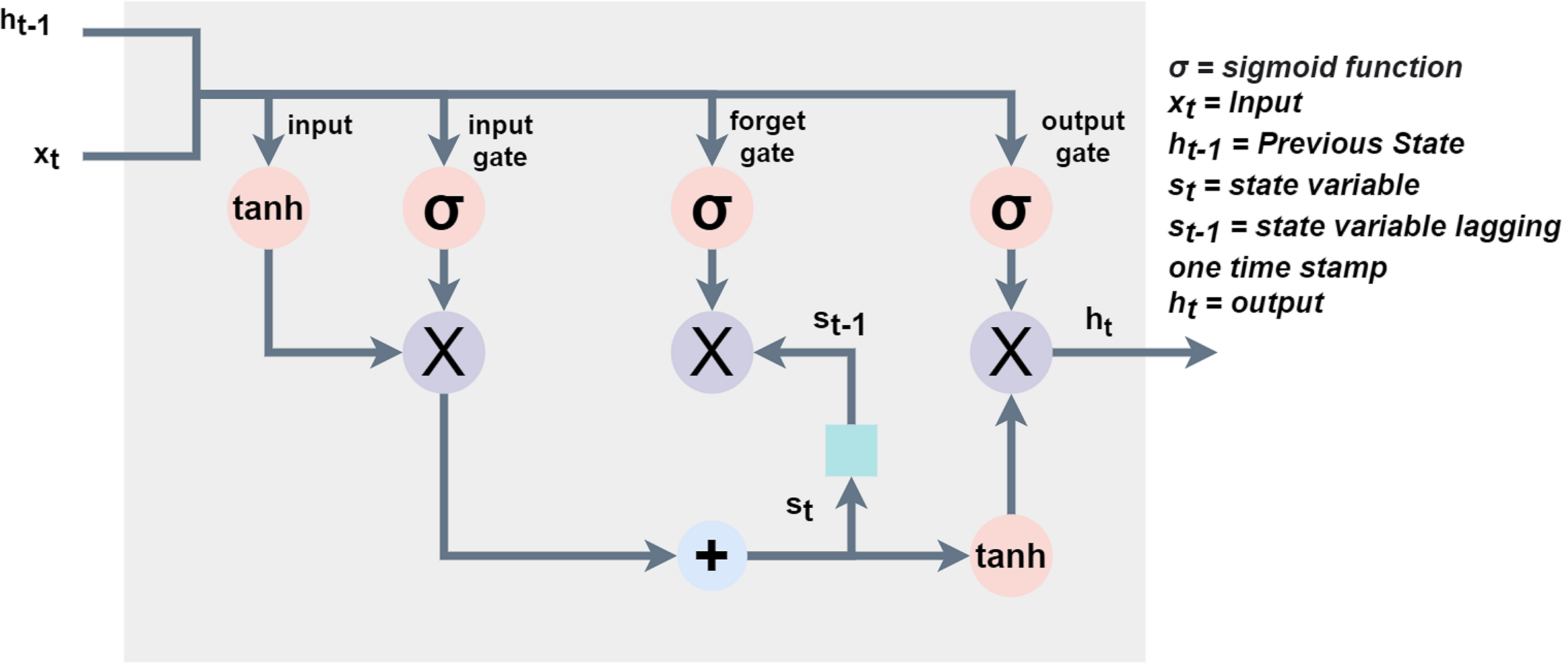
The architecture of the proposed model for sarcasm detection.

This study models sarcasm detection as a process of sequential labeling which takes an input as a sequence of embedded words. The model proposed in this study leverages the benefits of the dropout mechanism after embedding layer, one-dimensional CNN, and LSTM. Figure 3 shows the architecture of the proposed CNN-LSTM model.

The first module of our proposed framework is embedding which takes the input as a sequence of tokens and projects each token into a continuous low-dimension vector space. The embedding is initiated with arbitrary weights where the training set is fine-tuned to take embedding of all tokens as dense vectors of size *e*. In the proposed framework, the input size of the embedding layer is 1, 200-dimensional vector space and a vocabulary of 5,000 encoded integers ranging from 0 to 4,999. A dropout layer also called the regularization technique is used to restrict the embedded input from assimilating. The dropout layer arbitrarily drops some of the embeddings with a drop rate of 0.2 (Rao & Spasojevic, 2016). Utilizing the dropout layer on the embedded matrix helps to decrease the overfitting of deep neural networks (Sun & Gu, 2017). The rest of the word embedding which has not been dropped out are scaled as ${1 \over {1 - {p_e}}}$ where *p*_*e*_ is the likelihood of embedding dropout (Gal & Ghahramani, 2016).

Remaining of the word embedding after the dropout is then passed to the convolution module which performs 1-D convolution on the embedding rather than 2-D convolutions which are usually applied to the image data. In the 1-D convolution layer, a kernel is applied to the embedded input to map multiple features. Each neuron utilizes an activation function for learning non-linear features. In this study, the 1-D convolutional layer has a kernel of size 5 and 128 filters which reflects that 5-word combinations will be established by the filter thus considering combinations of words similar to the kernel size. To produce a non-linear relationship of mapped features the rectified linear unit (ReLU) is used as the activation function.

The output feature maps which represent regional features in a series of embedded words records the information in the feature map which is downscaled using the pooling mechanism (Zheng, Iwana & Uchida, 2019). A max-pooling layer has been integrated with this study which selects the maximum value observed in the kernel thus reducing the dimensionality of each input kernel into a single value. The pooling is utilized to retain the features with maximum presence in the dense vector space. It is worth mentioning that max-pooling remains constant to the significant pad tokens which are being added to the shorter sentences and retains the important information (Suárez-Paniagua & Segura-Bedmar, 2018). This study utilizes a pool size of four for max-pooling allowing the proposed framework to extract features with higher significance.

Lastly, the LSTM module has been explicitly chosen to overcome the gap of learning long-term dependencies. Here, an LSTM layer is used which utilizes the memory units instead of neurons and the neurons are set to 5,000 memory units. The blocks of the LSTM layer take max-pooled features as input, abstract them into a meaningful representation and utilize sigmoid as a default activation function. Followed by a dense layer, these features are then fed to the neurons of the fully connected network. The dense layer in the reference framework contains 1,000 neurons in which each neuron will further function to produce outputs for the next dropout layer. In the forward pass, the contribution of neurons is dropped at a rate of 0.2. The proposed network has a secondary dense layer set to one neuron and sigmoid as an activation function to integrate the input from previous layers to the final predictable output. The classification in this work is the binary classification which classifies the text as sarcastic or non-sarcastic. Each record in the multi-domain dataset is labeled as ’1’ for sarcastic and ’0’ for non-sarcastic. This study uses Adam optimizer for training while the number of the epoch is 50 and the batch size to 32.

### Feature extraction techniques used for experiments

For training and testing the proposed approach, three features are selected based on their popularity and wide use.

#### Term frequency-inverse document frequency

Term frequency-inverse document frequency (TF-IDF) is the most commonly used feature extraction technique for text analysis. Of the two important tasks of indexing and weighting for text analysis, TF-IDF deals with the weighting (Zhang, Yoshida & Tang, 2011). It finds the weight of a given term *t* in a given document *D*. TF-IDF is evolved from TF and IDF which are separate terms and can be calculated as

(1) $$TF(t) = \displaystyle{{{t_D}} \over {{N_D}}}$$

(2) $$IDF(t) = log\displaystyle{d \over {dt}}$$

where *t*_*D*_, *d* and *dt* represent the total number of *t* appearance in a document *D*, total number of documents and the number of documents that contain term *t*.

The weight of each term using the TF-IDF is computed through

(3) $${W_{t,d}} = T{F_{t,d}}\left( {\displaystyle{{{t_D}} \over {{d_{f,t}}}}} \right)$$

where *TF*_*t*,*d*_, and *d*_*f*,*t*_ represents the frequency of term *t* in document *d* and number of documents that contain *t*.

#### Bag of words

The bag of words (BoW) is a well-known and important feature extraction method that is widely used for text analysis, text classification, information retrieval, natural language processing, and topic modeling (Rustam et al., 2019; Liu et al., 2016). Besides being simple to implement and easy to interpret, it is often more resourceful than many sophisticated techniques. From the training *corpus*, it gathers the vocabulary of the unique words found in the text. It counts the occurrence of each unique word in a given text *corpus*. For the current study, the text is first tokenized and then the vocabulary is built which is later used to generate the vector. The generated vector is later used to train the proposed model.

#### Global vectors for word representation

Global vectors for word representation (GloVe) is a log-bilinear word embedding technique that is used to map the words in the form of vectors based on matrix factorization (Pennington, Socher & Manning, 2014). It is an unsupervised algorithm that converts the words into significant representations. The representations have semantic similarity concerning the distance among the words (Sharma et al., 2017). Being a count-based model, it is trained on the aggregated global word-to-word co-occurrence probabilities from a *corpus*. Additionally, GloVe performs dimensionality reduction to learn the vectors. The main objective of GloVe training is to learn the patterns of the word representations so that the probability of occurrence of the words becomes equal to the dot product of word representations (Si & Roberts, 2018). It then further generates the vectors based on their similarity and analogy tasks along with named entity recognition resulting in a large matrix of co-occurrence information. Words can be counted from the rows of the matrix and the frequency of words can be observed in the context through columns of the large *corpus*.

## Experiment and results

### Datasets used for experiments

Four datasets are used to evaluate the performance of both the selected machine learning classifiers and the proposed CNN-LSTM model. Two courses of action are followed for the evaluation where first the performance is analyzed on an individual dataset and then datasets are combined into a single dataset and results are analyzed. Single datasets are taken from multiple domains to comprehend the efficacy of the proposed approach. Similarly, the purpose of combining the dataset is to analyze the performance of the model on a combined multi-domain dataset. Four datasets are used in this study including the ‘Tweet’ dataset, ‘Reddit’ dataset, ‘Sarcasm *Corpus* V2’, and ‘News Headlines’ dataset.

**Figure 4:**
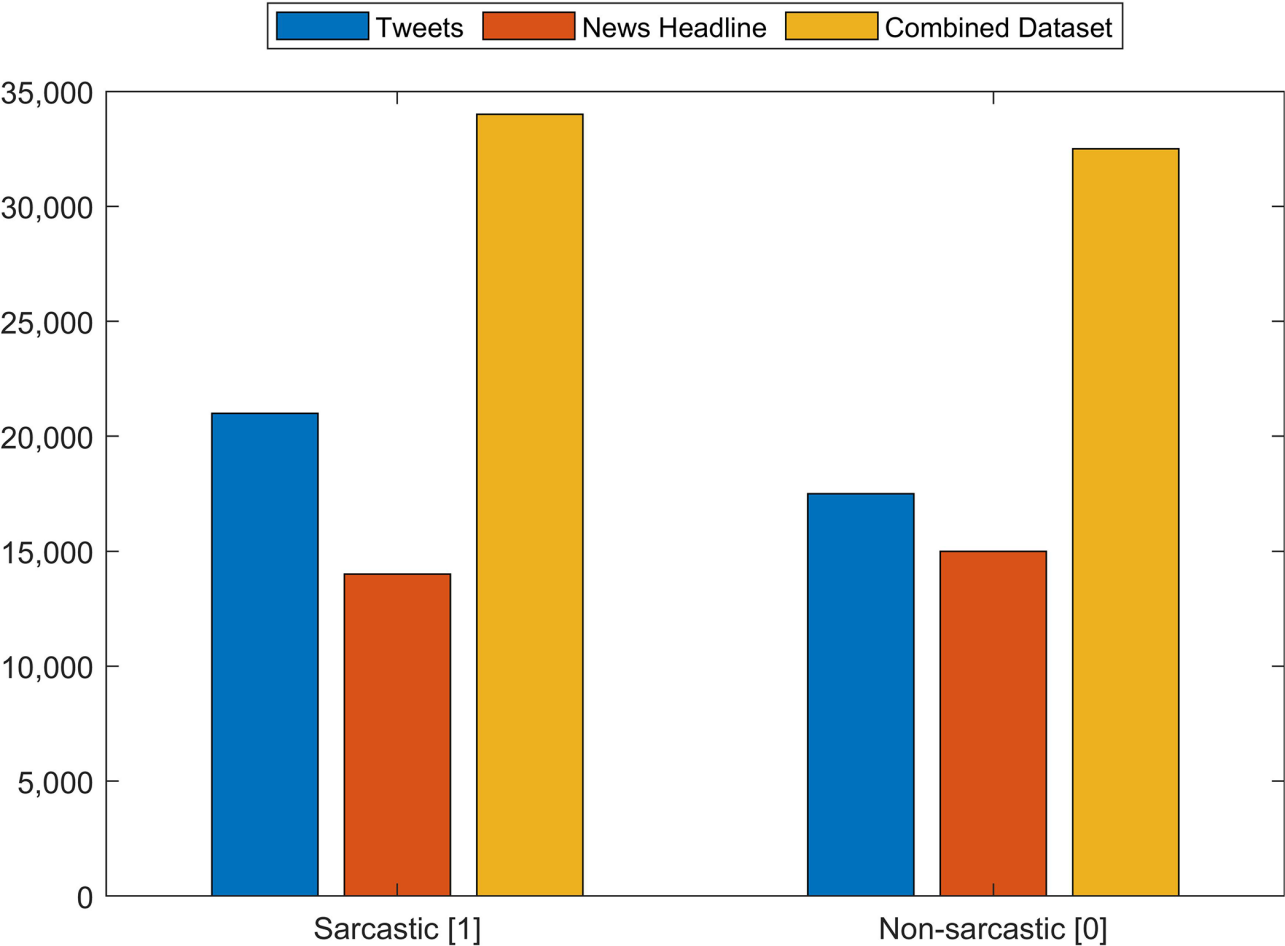
Number of sarcastic and non-sarcastic records in the Tweets dataset, the News Headlines dataset and a dataset constructed by merging these two datasets for training and testing.

The Tweet dataset contains 81,408 random tweets which are labeled as figurative (both sarcasm and irony), regular, sarcasm, and irony (John, 2020). From the tweets dataset, the tweets that are labeled sarcasm and regular are extracted which resulted in 39,267 tweets. Of the selected tweets, 20,681 are labeled as sarcasm while 18,595 as regular. The news headlines dataset contains 26,709 news headlines, which have been gathered from two different news websites ‘The Onion’ and ‘HuffPost’ (Misra, 2019). Among the news headlines, 14,985 headlines are labeled as sarcastic while 13,634 are non-sarcastic. We combine these two datasets (Tweets and News Headlines) to make a combined multi-domain dataset for training and testing of models. Figure 4 shows the distribution of sarcastic and non-sarcastic records for each dataset which are combined for testing. The details of the number of records in each dataset are given in Table 2. Combining the datasets into a single dataset provides 67,895 records which are used for the training and testing of models.

**Table 2:** Details for number of records in combined multi-domain dataset for training and testing.

Dataset	No. of records
Tweets	39,267
News headline	26,709
Total	67,895

**Figure 5:**
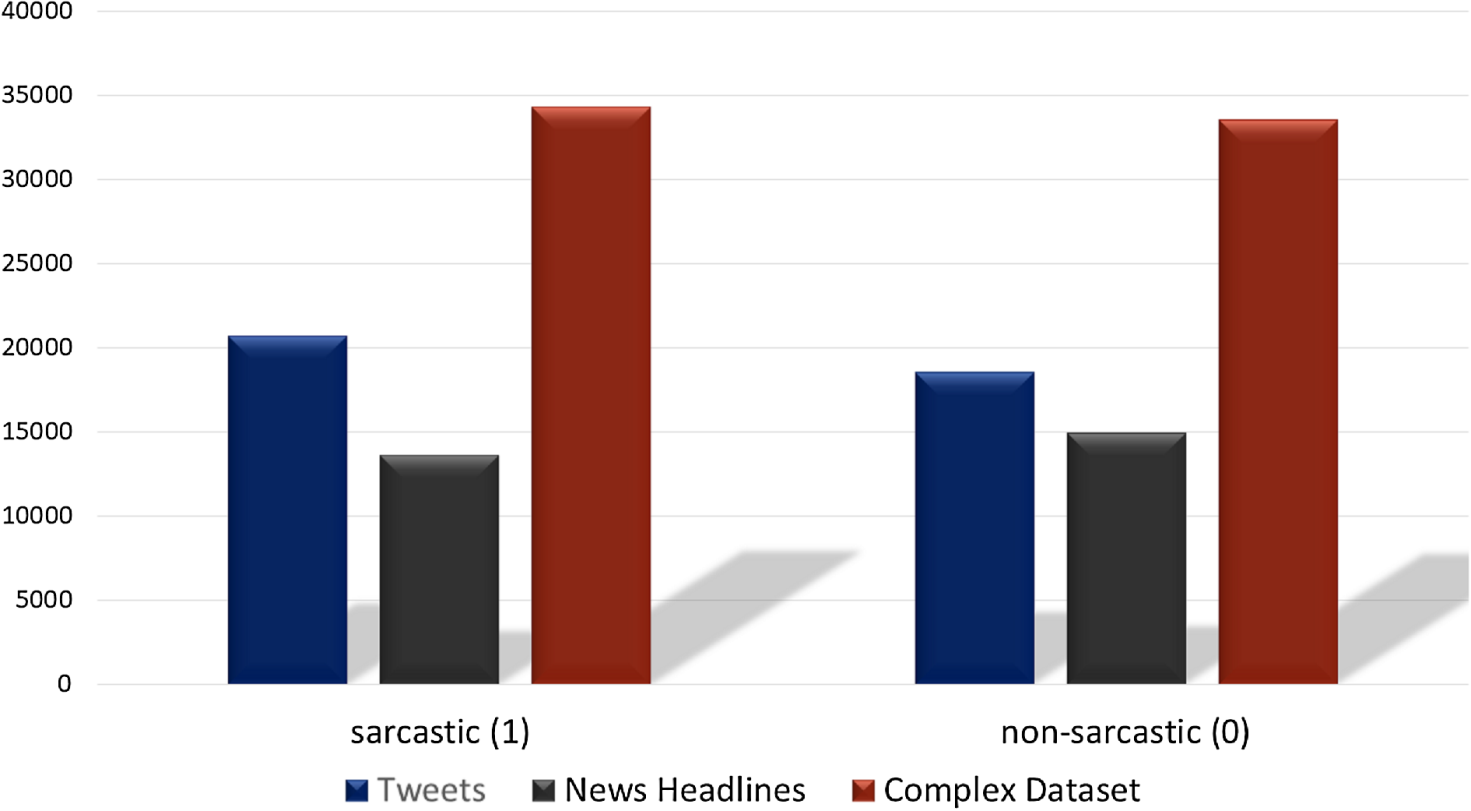
The distribution of sarcastic and non-sarcastic records for the combined multi-domain datasetused for validating the proposed CNN-LSTM model.

For the validation purpose, we combine two more datasets Reddit dataset and the Sarcasm *Corpus* V2 dataset. Reddit is a microblogging social media platform that contains news aggregation, user posts, discussion groups, and content ratings, etc. from various communities of people. The dataset is acquired from the Kaggle and contains sarcastic and non-sarcastic comments of users (Ofer, 2017). The fourth dataset is Sarcasm *Corpus* v2 which is a subset of ‘Internet Argument *Corpus*’ that contains posts annotated as sarcastic and non-sarcastic (Oraby et al., 2017). The combination of Reddit and Sarcasm *Corpus* V2 is used for the validation of models after training with combined (Tweets and News Headlines) multi-domain dataset. Figure 5 shows the distribution of sarcastic and non-sarcastic records for each dataset which are combined for validation. The details of the number of records in each dataset are given in Table 3. Combining the datasets into a single dataset result in 21,520 records which are used for the validation.

**Table 3:** Details for number of records in combined multi-domain dataset for validation.

Dataset	No. of records
Sarcasm *Corpus* V2	6,520
Reddit dataset	15,000
Total	21,520

### Experiment setup and performance evaluation metrics

**Figure 6:**
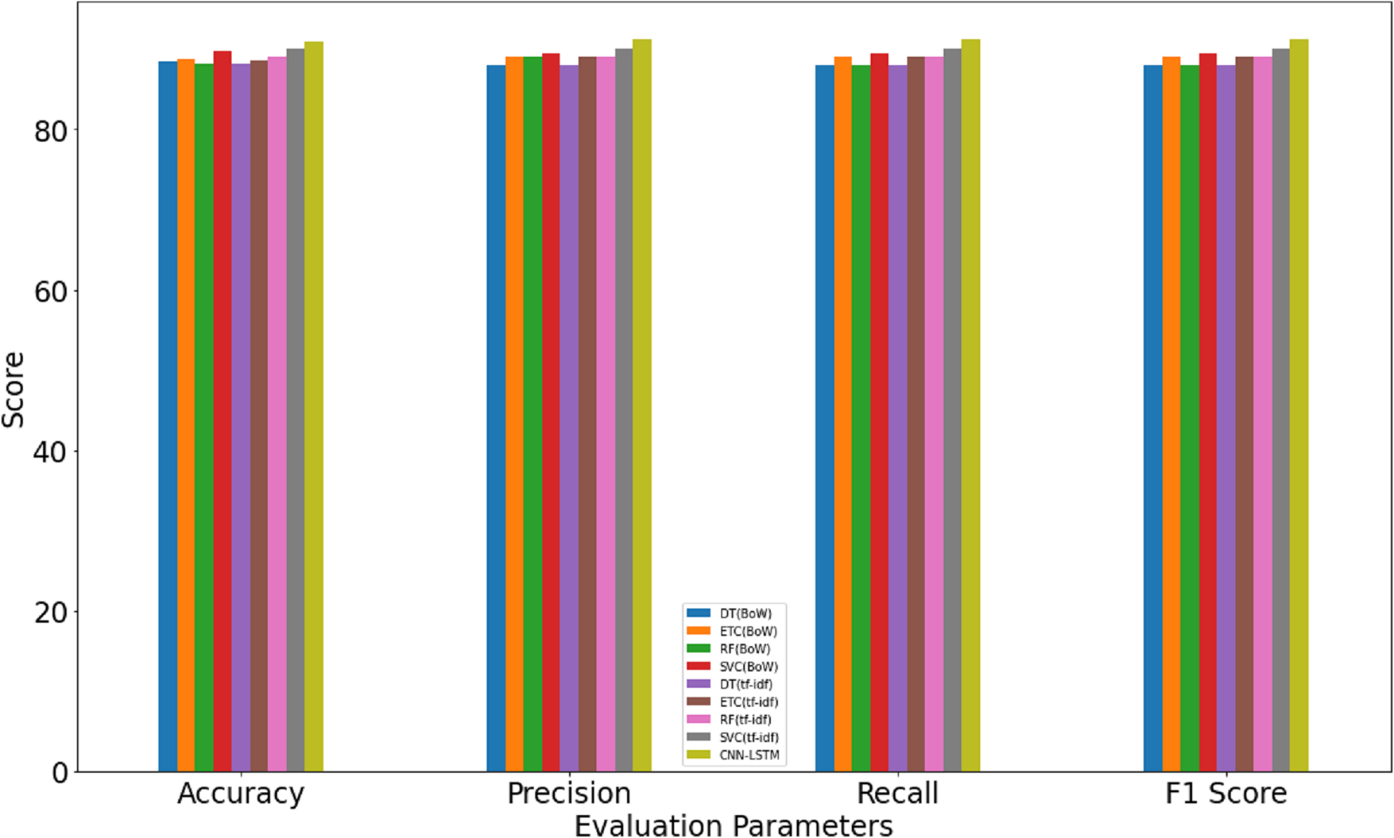
Framework of the proposed CNN-LSTM model for sarcasm detection.

The proposed approach is implemented in Python using TensorFlow which is a well-known interface for the development and execution of deep learning models (Ertam & Aydn, 2017). Figure 6 shows the flow of the proposed methodology used in the experiments.

The data used in this study is first preprocessed to minimize the computational overhead. For this purpose, the text data are converted into lowercase, numerical values and stop words are removed, and stemming is performed. Afterward, the data is split into train and test set into a 3:1 ratio. The training set is utilized for the training of the model whereas the model is tested on the test set. As previously described, three feature extraction techniques including TF-IDF, BoW and GloVe are utilized to extract features from the preprocessed data. For performance evaluation, the following metrics are adopted in this study.

Accuracy is one of the most commonly used performance evaluation measures for classification problems. The accuracy of a model is calculated as the ratio of the number of total correct predictions to the total number of predictions as follows

(4) $$Accuracy = \displaystyle{{TP + TN} \over {TP + TN + FP + FN}}$$

where

**TP (true positive)** is the text which is predicted as ‘sarcastic’ and it originally belongs to the ‘sarcastic’ class.

**TN (true negative):** is the text which originally belongs to the ‘non-sarcastic class and the model predicts it so.

**FP (false positive):** is the text which is predicted as ‘sarcastic’ but it originally belongs to the ‘non-sarcastic’ class.

**FN (false negative):** is the text which is incorrectly predicted as ‘non-sarcastic’ while it originally belongs to the ’sarcastic’ class.

Precision and recall are two important metrics and represent a model’s capability to make precise and sensitive predictions. Following equations are used for precision and recall

(5) $$Precision = \displaystyle{{TP} \over {TP + FP}}$$

(6) $$Recall = \displaystyle{{TP} \over {TP + FN}}$$

Precision represents the ratio of correctly predicted positive instances to total predicted positive instances. Precision relates to the false positive rate and high precision indicates the low false positive rate. Recall also called sensitivity refers to the ratio of corrected predicted positive instances to all positive instances in the class.

F-Score considers both precision and recall and is considered more appropriate than precision and recall alone. *F*_1_ score is a function of precision and recall and can be calculated as

(7) $${F_1}Score = 2 \times \displaystyle{{Precision \times Recall} \over {Precision + Recall}}$$

### Results and discussions

Several experiments are performed using machine learning algorithms and three different feature extraction approaches. TF-IDF, BoW, and GloVe are used for feature extraction with the machine learning classifiers while the proposed approach is directly trained on the Sarcasm *Corpus* V2 dataset.

#### Results using single domain dataset

Initially, the experiments are performed using a single dataset that contains the sarcastic and non-sarcastic records from a single domain only. For this purpose, the Tweets dataset is used to evaluate the performance of the machine learning classifiers, as well as, the proposed CNN-LSTM model. Results are shown in Table 4. Results indicate that the RF, ETC achieves the highest accuracy among the machine learning classifiers with an accuracy of 0.85 each while the proposed CNN-LSTM model correctly predicts 92% of the sarcastic and non-sarcastic text. As discussed in related work, the performance of various machine learning and other proposed models is good with a single domain dataset; however, these models are not tested with the multi-domain dataset. The main objective of this study is to analyze the impact of data complexity and multi-domain dataset on classification accuracy. Hence, several experiments are performed to investigate the accuracy of the CNN-LSTM model with the combined multi-domain dataset.

**Table 4:** Results using the Tweets dataset and machine learning classifiers using TF-IDF.

Model	Accuracy	Class	Precision	Recall	F1 score
CNN-LSTM	0.92	Sarcasm	0.93	0.92	0.92
		Non-sarcasm	0.92	0.92	0.92
		Macro Avg	0.92	0.92	0.92
RF	0.85	Sarcasm	0.88	0.85	0.86
		Non-sarcasm	0.84	0.83	0.83
		Macro Avg	0.86	0.84	0.84
ETC	0.85	Sarcasm	0.87	0.85	0.86
		Non-sarcasm	0.83	0.85	0.84
		Macro Avg	0.85	0.85	0.85
SVC	0.81	Sarcasm	0.81	0.81	0.81
		Non-sarcasm	0.81	0.79	0.80
		Macro Avg	0.81	0.80	0.81
DT	0.83	Sarcasm	0.83	0.83	0.83
		Non-sarcasm	0.83	0.82	0.82
		Macro Avg	0.83	0.83	0.83

#### Testing results using TF-IDF, BoW and GloVe using multi-domain dataset (Tweets and News Headlines)

Each term’s frequency score within the document and across all the other documents in a *corpus* is computed to retrieve the relevant and vital features. Similarly, BoW and GloVe are used to extract features from the text *corpus*. After that, the extracted features are fed to the machine learning models including DT, RF, ETC, and SVC for training and classification. Experimental results for the machine learning classifiers are shown in Tables 5, 6 and 7.

**Table 5:** Experimental results for machine learning classifiers using BoW features.

Model	Accuracy	Class	Precision	Recall	F1 score
RF	0.88	Sarcasm	0.90	0.88	0.89
		Non-sarcasm	0.88	0.87	0.87
		Macro Avg	0.89	0.88	0.88
ETC	0.88	Sarcasm	0.89	0.91	0.90
		Non-sarcasm	0.88	0.87	0.88
		Macro Avg	0.89	0.89	0.89
SVC	0.89	Sarcasm	0.91	0.90	0.90
		Non-sarcasm	0.89	0.89	0.89
		Macro Avg	0.90	0.90	0.90
DT	0.88	Sarcasm	0.88	0.88	0.88
		Non-sarcasm	0.88	0.88	0.88
		Macro Avg	0.88	0.88	0.88

**Table 6:** Experimental results for machine learning classifiers using TF-IDF features.

Model	Accuracy	Class	Precision	Recall	F1 score
RF	0.89	Sarcasm	0.89	0.89	0.89
		Non-sarcasm	0.89	0.89	0.89
		Macro Avg	0.89	0.89	0.89
ETC	0.88	Sarcasm	0.89	0.89	0.89
		Non-sarcasm	0.89	0.88	0.88
		Macro Avg	0.89	0.89	0.89
SVC	0.90	Sarcasm	0.90	0.90	0.90
		Non-sarcasm	0.90	0.90	0.90
		Macro Avg	0.90	0.90	0.90
DT	0.88	Sarcasm	0.88	0.88	0.88
		Non-sarcasm	0.88	0.88	0.88
		Macro Avg	0.88	0.88	0.88

**Table 7:** Experimental results for machine learning classifiers using GloVe features.

Model	Accuracy	Class	Precision	Recall	F1 score
RF	0.81	Sarcasm	0.83	0.81	0.82
		Non-sarcasm	0.78	0.79	0.78
		Macro Avg	0.81	0.80	0.80
ETC	0.81	Sarcasm	0.81	0.81	0.81
		Non-sarcasm	0.81	0.80	0.80
		Macro Avg	0.81	0.81	0.81
SVC	30.76	Sarcasm	0.76	0.76	0.76
		Non-sarcasm	0.76	0.76	0.76
		Macro Avg	0.76	0.76	0.76
DT	0.68	Sarcasm	0.69	0.68	0.68
		Non-sarcasm	0.67	0.68	0.67
		Macro Avg	0.68	0.68	0.68

Results indicate that the machine learning classifiers perform better when used with BoW and TF-IDF features than that of GloVe features. SVC achieves the highest accuracy of 0.90 and 0.89 for TF-IDF and BoW, respectively while RF, DT, ETC have slightly low accuracy. The scores for precision, recall, and F score is following the accuracy. Testing results using the proposed CNN-LSTM model, as given in Table 8, suggest that it surpasses the accuracy of machine learning classifiers with an accuracy score of 0.916 which is higher than other classifiers.

**Table 8:** Performance of the proposed CNN-LSTM model.

Model	Accuracy	Class	Precision	Recall	F1 score
RF	0.916	Sarcasm	0.93	0.92	0.92
		Non-sarcasm	0.89	0.90	0.90
		Macro Avg	0.91	0.91	0.91

The performance of machine learning models with BoW and TF-IDF features is better as compared to GloVe because BoW and TFIDF give simple term frequency and weighted feature vectors, respectively. On the other hand, GloVe generates a co-occurrence matrix as a feature set that is complex and large than both BoW and TF-IDF features sets. As a result, models do not perform well with GloVe features. Similar results are reported in Tabashum et al. (2020). However, the performance of feature extraction methods can vary concerning the nature of the dataset used for experiments. For example, GloVe is reported to show better performance than TF-IDF for the sparse dataset in Dorani, Duru & Yildiz (2019). However, for the datasets, where the class samples are non-sparse, the performance of TF-IDF is better than the GloVe features.

The performance of CNN-LSTM exceeds all the other models as neural networks can store context information temporarily at each step forward that enables it to perform better than the conventional machine learning algorithms. Therefore, CNN is applied to map multiple features on the embedded input, each neuron utilizes an activation function to learn non-linear features, and LSTM is used to overcome the gap of learning long-term dependencies. For a clear understanding of the performance of various classifiers, the total number of TP, TN, FP, and FN, as well as, correct predictions (CP) and wrong predictions (WP) are given in Table 9. The correct predictions are derived by adding TP and TN while wrong predictions comprise FP and FN.

**Table 9:** Prediction results for machine learning algorithms and CNN-LSTM.

Models	TP	TN	FP	FN	CP	WP
**BoW**						
DT	7344	7660	917	1053	15004	1970
RF	7823	7144	438	1569	14967	2007
ETC	7768	7298	493	1415	15066	1908
SVC	7558	7697	703	1016	15255	1719
**TF-IDF**						
DT	7357	7608	904	1105	14965	2009
RF	7754	7370	507	1343	15124	1850
ETC	7189	7845	1072	868	15034	1940
SVC	7549	7782	712	931	15331	1643
**GloVe**						
DT	5777	5693	2551	2953	11470	5504
RF	6881	6774	1447	1872	13655	3319
ETC	6776	6890	1552	1756	13666	3308
SVC	6416	6466	1912	2180	12882	4092
**CNN features**						
CNN-LSTM	7689	7698	724	863	15387	1587

Experimental results given in Table 9 show that the proposed CNN-LSTM has given state-of-the-art results on a multi-domain dataset because of its robustness compared to the other machine learning models. The number of correct predictions of each model can be determined and it can be observed that the CNN-LSTM has the minimum number of FP, and FN predictions and has predicted the maximum number of TP and TN predictions. Its ability to save context information for a short period to map long-term dependencies helps it to make more accurate predictions than any other machine learning model. Furthermore, its ability to drop out the least useful data helped to achieve better performance. In particular, it has the highest rate of correct predictions on the test data containing 15,387 records. Of the 1,587 wrong predictions from CNN-LSTM, 724 are non-sarcastic and 863 are sarcastic.

The performance of the proposed model is superior due to several important factors. The logical structure of the proposed model enables it to achieve relatively better performance. A one-dimensional CNN is used to map features from multi-domain data. CNN uses weights to learn from input, each neuron uses activation functions to learn non-linear features from the data. The ReLU is used as the activation function to produce a non-linear relationship of the mapped features. Then, LSTM is used to overcome the gap of long-term dependencies. LSTM uses memory units and takes max-pooled features as input and extracts them into meaningful representation. Neural networks can learn imperative features from data automatically which enables the proposed model to learn the hidden relationship carried by sarcastic records. The architecture of the proposed model helps to learn the complex relationship and improves the prediction accuracy.

#### Validation results using TF-IDF, BoW and GloVe using multi-domain dataset (Reddit and Sarcasm *Corpus* V2)

The validation of the proposed approach is performed using a combined dataset that comprises the results from three datasets from different domains. The combined dataset combines the records from the Reddit and Sarcasm V2 datasets to make a combined dataset and the distribution of sarcastic and non-sarcastic records is shown in Fig. 5.

Experimental results given in Table 10 indicate that the classification accuracy, as well as, other performance evaluation measures have been degraded when machine learning algorithms are validated on the combined dataset. The primary reason for such performance decrease is the complex nature of the dataset which contains the data from multiple domains. The classifiers are trained and validated on different datasets wherein the training data is from tweets, and news headlines only while the validation data contains the records from Reddit and Sarcasm V2 in addition to News Headlines. The change in the training and validation data makes it very challenging for the trained classifiers to make accurate predictions. Consequently, the accuracy of SVC which had a leading performance with the testing data by achieving 0.90 accuracies with TF-IDF has reduced to an accuracy of 0.70 with TF-IDF. The performance of the other classifiers is affected in a similar proportion.

**Table 10:** Results for machine learning classifiers on the combined validation dataset.

Parameters	BoW				TF-IDF				GloVe			
	DT	RF	ETC	SVC	DT	RF	ETC	SVC	DT	RF	ETC	SVC
Accuracy	0.60	0.68	0.68	0.69	0.62	0.70	0.70	0.70	0.59	0.66	0.66	0.60
Precision	0.69	0.70	0.67	0.68	0.66	0.71	0.70	0.69	0.58	0.66	0.66	0.60
Recall	0.55	0.65	0.66	0.68	0.55	0.67	0.68	0.69	0.58	0.64	0.64	0.56
F-Score	0.47	0.64	0.66	0.68	0.49	0.67	0.68	0.69	0.58	0.64	0.64	0.53

The performance of the proposed CNN-LSTM using the combined validation dataset is shown in Table 11. It can be seen that it can achieve a classification accuracy of 0.73 with the combined dataset even when the highest accuracy of the machine learning classifiers is 0.70. Despite the reduction due to the dataset complexity, CNN-LSTM still performs better than other classifiers. It indicates the superiority of the proposed CNN-LSTM on the multi-domain dataset. It also suggests that the proposed CNN-LSTM can be potentially used for sarcasm detection on the multi-domain dataset.

**Table 11:** Performance of the proposed CNN-LSTM model on the validation dataset.

Parameters	Proposed CNN-LSTM
Accuracy	0.73
Precision	0.74
Recall	0.72
F1-Score	0.73

### Performance comparison with state-of-the-art approaches

The performance of the proposed model is analyzed against several state-of-the-art approaches for sarcasm detection. For a fair comparison, the selected models are implemented and tested on the multi-domain dataset (Tweets and News Headlines; Table 2). Table 12 shows the comparison of experimental results of these models. The results showed that the proposed approach performs significantly better than the state-of-the-art approaches in detecting sarcastic tweets from the combined multi-domain dataset.

**Table 12:** Performance analysis of the proposed approach with state-of-the-art approaches.

Reference	Techniques	Accuracy
(Gupta et al., 2020)	Chi2, TFIDF, Voting classifier	89%
(Khatri, Pranav & Anand, 2020)	GloVe, with logistic regression	86%
(Majumder et al., 2019)	GRU-based neural network	90%
(Mandal & Mahto, 2019)	Deep CNN-LSTM	86%
Current study	CNN-LSTM	91.6%

## Conclusion

Sarcasm detection has been one of the most widely researched areas during the last few years due to the expansion and large use of social media platforms. However, predominantly, such works focus on the single domain datasets and show good performance. Multi-domain datasets, on the other hand, are complex and pose a real challenge. This study presents a CNN-LSTM model to cope with the challenge of sarcasm detection in the multi-domain dataset. Four datasets are used in this study including Tweets, News Headlines, Reddit, and Sarcasm *Corpus* V2. Also, different training and validation datasets are used where training is carried out on the combined dataset of Tweets and News Headlines while the validation is done on the combined dataset of News Headlines, Reddit and Sarcasm V2. Testing results show an accuracy of 0.916 using the proposed CNN-LSTM than the 0.90 accuracy of SVC from the machine learning classifiers. Validation on multi-domain dataset tend to decrease the performance of all the classifiers and the accuracy is reduced to 0.70 and 0.73 fro the SVC and CNN-LSTM, respectively. The results suggest that the proposed CNN-LSTM has the potential to perform sarcasm detection on the combined multi-domain dataset and produce competitive results to that of state-of-the-art approaches that work on single domain datasets.

## Supplemental Information

10.7717/peerj-cs.645/supp-1 Supplemental Information 1 Implementation code.

## Additional Information and Declarations

### Data Availability

The following information was supplied regarding data availability: Python implementations for the model are available in the Supplemental File.
